# Achieving stable remission with maintenance electroconvulsive therapy in a patient with treatment-resistant schizophrenia: A case report

**DOI:** 10.1097/MD.0000000000008813

**Published:** 2017-12-01

**Authors:** Sebastian Moeller, Neele Kalkwarf, Caroline Lücke, Diana Ortiz, Sonja Jahn, Christiane Först, Niclas Braun, Alexandra Philipsen, Helge H.O. Müller

**Affiliations:** a Medical Campus University of Oldenburg, School of Medicine and Health Sciences, Psychiatry and Psychotherapy—University Hospital; b Geriatric Psychiatry; c ECT Unit, Karl-Jaspers-Klinik, Bad Zwischenahn, Germany.

**Keywords:** electroconvulsive therapy, maintenance electroconvulsive therapy, neurostimulation, remission, treatment-resistant schizophrenia

## Abstract

**Rationale:**

Up to one third of all schizophrenic patients are classified as having treatment-resistant schizophrenia (TRS). This subgroup faces remarkable medical and psychosocial damages, and pharmacotherapy is often limited due to nonresponse and/or side effects. Maintenance electroconvulsive therapy (M-ECT) might be effective in TRS.

**Patient concerns:**

We present a case of a 26-year-old male patient with a TRS.

**Diagnoses:**

He received a treatment series of ECT sessions and a course of 24 M-ECTs.

**Interventions:**

The entire treatment was tolerated without significant side effects.

**Outcomes:**

Moreover, the Psychotic Symptom Rating Scale (PSYRATS) scores for both positive and negative symptoms decreased and remained stable over the course of M-ECT.

**Lessons:**

Because of the remarkable improvement in the negative and positive symptom clusters, we propose systematic examinations in the field of M-ECT in TRS patients. These studies should integrate long-term outcome and tolerance measurements, gaining insight into the optimal duration of treatment for this indication.

## 1 Background

Up to one third of patients with schizophrenia are classified as having treatment-resistant schizophrenia (TRS),^[1–6]^ the diagnostic criteria for which^[7–11]^ include lack of response to 2 different antipsychotic trials or clozapine, intolerance of antipsychotic drug side effects, and relapse or symptomatic deterioration even when taking sufficient doses of the appropriate medication.^[12–15]^ Other widely accepted criteria of TRS include an illness duration of >5 years; psychotic-associated symptoms that show no significant improvement after 2 years of regular, full-dose/full-course treatment with 2 kinds of antipsychotics; and, especially, no response to clozapine.^[16–23]^

Although clozapine is the criterion standard for the treatment of patients with TRS, clinical symptoms persist in approximately 40% to 70% of clozapine users.^[22,24–26]^ For TRS and/or clozapine non- or partial response in TRS, in addition to a variety of pharmacological and nonpharmacological approaches, electroconvulsive therapy (ECT) has been attempted as an adjunct therapy for schizophrenia, especially when a rapid improvement and symptom reduction is desired.^[20,27–31]^ ECT, first developed in the 1930s by Bini and Cerletti, is a key neurostimulation tool within the therapeutic armamentarium in clinical psychiatry for severe and life-threatening psychiatric diseases, particularly depressive diseases but also in cases of TRS.^[32]^ In an extensive Cochrane review on randomized controlled trials (RCTs) comparing real and sham ECT for schizophrenia, a larger improvement was found for those patients that received real ECT.^[33–36]^ Moreover, several studies have demonstrated the efficacy of ECT for patients with TRS. Among other things, fewer relapses and a greater likelihood of an earlier discharge from the hospital were observed after ECT.^[27,37–39]^ Moreover, a longer treatment series with 20 electroconvulsive treatments was more efficient than a shorter treatment series with 12 treatments. Hence, these findings indicate that ECT might be a valuable adjunct therapy to antipsychotic medication in TRS.^[40–42]^

Although ECT might be considered a promising adjunct therapy for TRS, there are insufficient empirical data on the duration of the beneficial antipsychotic effects of ECT. In fact, no controlled RCTs have investigated ECT maintenance treatment (M-ECT) in TRS. M-ECT characterizes the maintenance treatment after successful treatment of an index phase, mostly in major depression and especially in depression with psychotic symptoms. However, M-ECT might also be effective in TRS.^[43]^

Here, we present the case of a 26-year-old chronic psychotic patient who improved remarkably and whose psychotic symptoms remained stable after continuously receiving M-ECT.

## 2 Case presentation

The 26-year-old male patient had been followed-up for schizophrenia for approximately 3 years after suffering from fluctuating paranoid-hallucinatory symptoms since he was 20 years old. Therefore, the diagnosis of paranoid schizophrenia was made by multiple psychiatrists. No family history of neurological or psychological illness was identified. Despite taking neuroleptics, in the course of the disease, the patient experienced 4 episodes (for several weeks) with paranoid-hallucinatory exacerbation. In these episodes, the patient suffered from formal thought disorders, fear, and delusions of persecution; auditory hallucinations with commenting, discussing, and commanding voices; visual hallucinations involving seeing persons in his room; and tactile hallucinations with the feeling of being touched from behind. In parallel to these exacerbations, the patient developed severe negative and cognitive symptoms including attention and memory deficits, fatigue, depressive mood, and sleep disturbances, and thus completing the psychopathological features of comprehensive schizophrenia. Neurological and medical examinations showed no further clinical disorders. The patient had been treated with amisulpride, benperidol, chlorprothixene, levomepromazine, olanzapine, and clozapine at standard doses and for an adequate period of time. Because he did not properly response to any of these medications, we diagnosed him as having TRS and initiated an individual treatment regime with M-ECT. In total, 24 ECT treatments were administered over a 1-year period, and the treatment success was evaluated by the Psychotic Symptom Rating Scales (PSYRATS), (Table 1).^[44,45]^ Before beginning ECT treatment, the patient showed clear deficits in almost every PSYRATS subscale; however, after 24 ECT treatment sessions, the symptom severity was much weaker and limited to only some of the PSYRATS subscales (see tables). His neuroleptic medication, olanzapine (10 mg given morning and night), remained stable during the treatment course. Moreover, there was no need for add-on psychiatric in-house treatment during the whole M-ECT treatment course (Table 2).

**Table 1:**
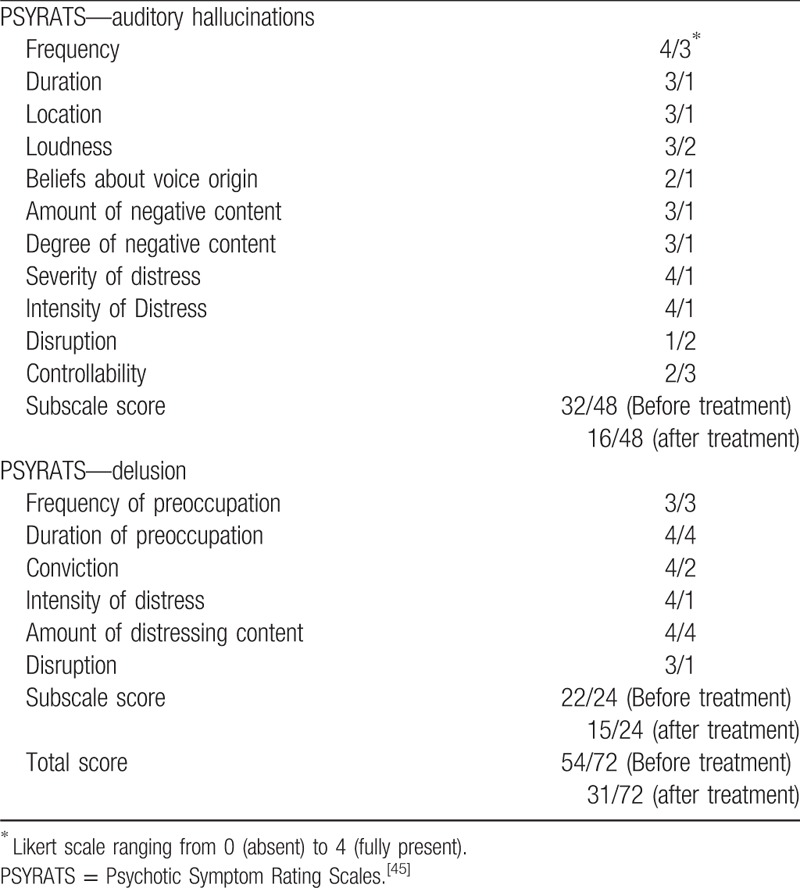
Psychotic Symptom Rating Scale scores before and after maintenance electroconvulsive therapy.

**Table 2:**
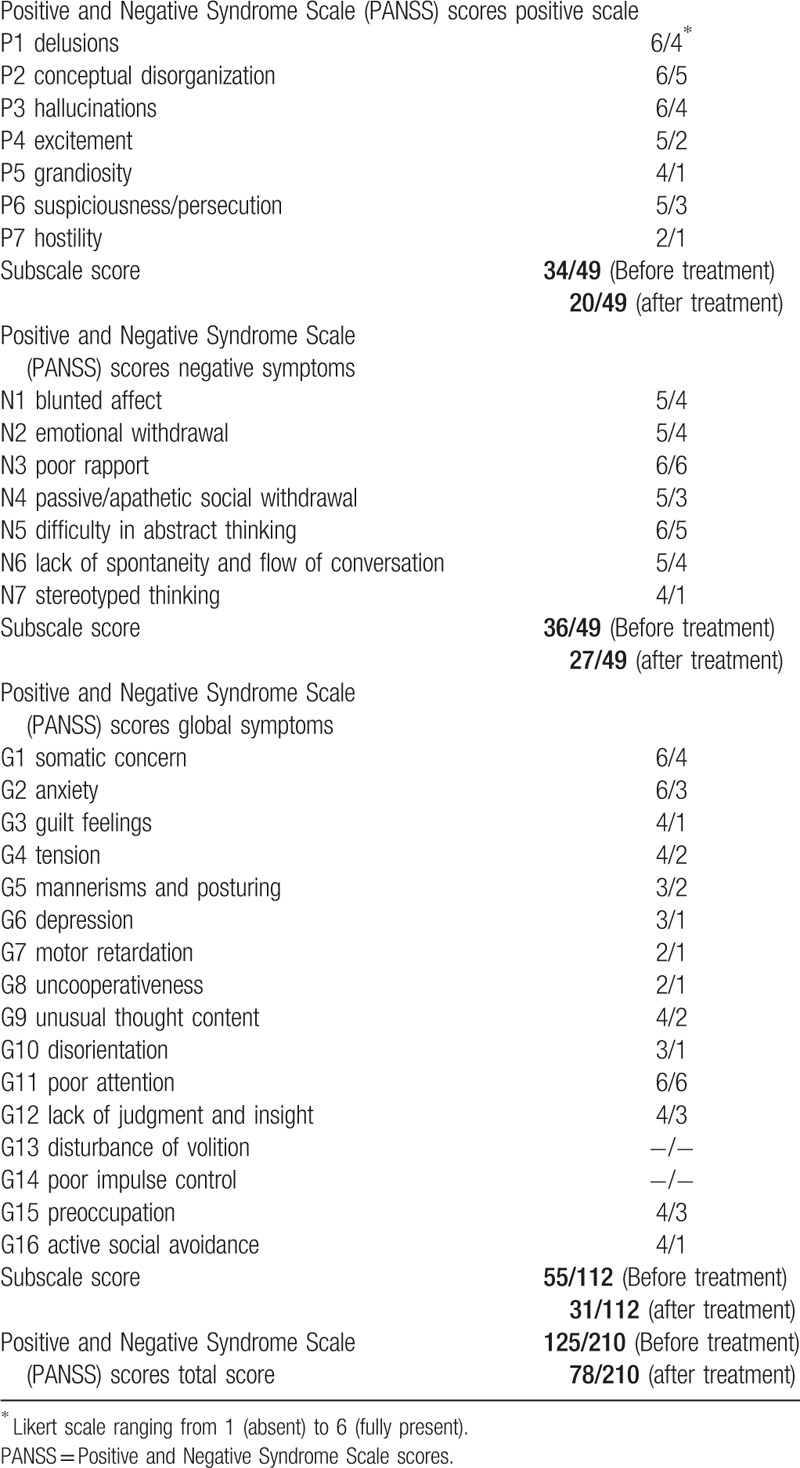
Positive and Negative Syndrome Scale scores before and after electroconvulsive therapy.

## 3 Discussion

Treatment of TRS is generally challenging. Our patient with TRS received different neuroleptic drugs at standard doses for an adequate period of time, none of which led to a satisfactory outcome as the psychotic symptoms persisted. The patient's improvement exactly corresponded to the initiation of M-ECT. Moreover, his psychotic symptoms remained stable throughout the course of M-ECT. Despite the symptom reduction, the patient reported a good tolerance and compliance for the M-ECT treatment course. Furthermore, the total and subscale scores of the PSYRATS changed for the better. Our patient's positive symptoms, that is, delusion and hallucinations, as well as his negative symptoms, that is, blunted affect and emotional withdrawal, improved. Of upmost importance, even a stay at a forensic psychiatry clinic and aggressive tendencies could be prevented due to the treatment.

Although the use of ECT in acute or even life-threatening phases of mental illnesses, for example, catatonic conditions, is well known and evidence-based, there is a lack of information regarding M-ECT in chronic and nonresponsive schizophrenic probands.^[40–43]^

Our case demonstrates that M-ECT might be a promising option to reduce the likelihood of new psychotic episodes.^[43]^ Moreover, in line with existing literature,^[31]^ the patient did not report any side effects, for example, memory impairment, probably because, unlike acute ECT, the time interval between treatments is longer.^[43]^ However, there is no agreement in the literature on the optimal duration of M-ECT treatment in TRS cases.

Our patient received bilateral M-ECT under general anesthesia. Initially, we performed ECT once a week; later we performed it once every second week followed by ECT once in a month. Because there is not yet a general consensus on the frequency of M-ECT therapy, mostly flexible and individually scheduled weekly/biweekly/monthly courses are used.^[43]^

We only report 1 single case and thus cannot completely rule out the possibility that our patient's improvement was independent of the acute and maintenance ECT but was rather a spontaneous remission or unspecific adherence-based effect, for example, frequent welfare during his stays at the hospital. Moreover, the narcosis during ECT might also have influenced the symptoms of our patient.^[46]^ However, the improvement seen in our patient seems to be clearly associated with M-ECT. We thus propose systematic examinations in that field.

To conclude, our case demonstrates the efficacy and safety of M-ECT in a patient with TRS who showed a significant improvement in terms of his positive and negative symptoms. Larger studies should not only examine outcome measurements but also integrate questions of tolerance and the duration of treatment courses.
